# Supplementary material for: Estimation of long-term river discharge and benefits evaluation of ecological water conveyance in Ertanggou basin from 2000 to 2020
Source: PLoS One. 2025 May 9;20(5):e0321990. doi: 10.1371/journal.pone.0321990 (PMC12063809; doi:10.1371/journal.pone.0321990)
Supplement: Attachment — Submitted filename: Response to Reviewers.docx [file pone.0321990.s002.docx]

To Editor:

Thank you for your letter and the constructive comments on this article in your busy schedule. All of us authors have carefully read the comments that you have given us, and have discussed and revised each of these issues. The following is my list of revisions. In addition, we have resubmitted a new manuscript in the revised state, with the revisions highlighted in red. If there are any incorrect answers or questions in the manuscript, please do not hesitate to let us know.

Kind regards,

Adilai Wufu

**Response letter to Reviewer 1**

**(our responses are in blue color and the actions in reviewed manuscript are in red color)**

We are very appreciated the time and effort you have put into your comments. Your advice about the upgrading of the data, structure and references of my paper is most helpful. I’ve made the necessary revisions, I have the paper professionally proofread to ensure that I’ve met all the requirements consistently, all of the comments were revised very carefully as you expected.

**Comment1**: They should use the observed field discharge from secondary sources to make sure about the actual discharge happening in the region that is missing.

**Answer:** Thank you for raising this concern regarding data reliability. Our study area, a small river basin, lacks established hydrological stations and official data from relevant water resources authorities. To validate our discharge estimates, we compared the measured flow velocity and discharge with results from the RSHS estimation model. The RMSE values for velocity and discharge were 0.2 m/s and 3.2 m³/s, respectively, with NSE values of 0.97 and 0.94, demonstrating high reliability. Additionally, the relative accuracy values of all estimates were within 20%, meeting the "Hydrological Information and Forecasting Standards" (GB/T 22482-2008) by China’s water resources department.

While secondary data would have been ideal, its absence necessitated reliance on field measurements and analytical methods, which we believe provide a solid foundation for validating our model. The data results are shown in Table 3.

These results are detailed in Section 3.1.2 of the manuscript.

**Table 3.** Relative accuracy evaluation table of flow velocity and discharge

| Section | Measured  flow velocity  (m/s) | Estimate  flow velocity  (m/s) | RA (%) | Section | Measured  discharge  (m³/s) | Estimate  discharge  (m³/s) | RA (%) |
| --- | --- | --- | --- | --- | --- | --- | --- |
| K1 | 1.35 | 1.57 | 16.3 | K1 | 32.1 | 25.76 | 19.8 |
| K2 | 4.1 | 3.92 | 4.4 | K2 | 2.8 | 2.3 | 17.9 |
| K3 | 2.1 | 1.86 | 11.4 | K3 | 2.2 | 1.92 | 12.7 |
| K4 | 1.15 | 1.31 | 13.9 | K4 | 2.35 | 2.71 | 15.3 |

**Comment2**: From 2000 to 2020, the river discharge of the Ertanggou basin in Xinjiang grew overall. From 2016 to 2020, the discharge of the K1 portion upstream of the reservoir was much higher than that of the other sections, primarily driven by river enhancement and the interception effect, that significantly enhance water resource efficiency and decrease waste. But in previous years the discharge remains same why it is so? Has not been explained and also discharge peaked only in 2 years and again it has come back to the same level what is it meant for glacier melt that is linked to CC. In other location as well even though discharge is less as expected there is no variation in last two decades what may be the reason, they have simplified their observation it sems and it needs intense discussion here.

**Answer:** Thank you for highlighting these observations. To address this, we have revised the manuscript to include a more in-depth discussion.

The relatively stable discharge before 2016 is attributed to the natural variability of glacial meltwater, the primary water source for the river ^[24]^. The rate of glacier melt showed a stable, slow growth trend during this period, resulting in limited changes in water input to the basin. The observed peaks in discharge, such as in 2018, correspond to temperature increases across Xinjiang, which accelerated glacier melt. However, these peaks are temporary and reflect the seasonal nature of glacial contributions ^[25]^.

The minimal variation in discharge at downstream sections over two decades is due to geographical and usage factors. Upstream areas, situated at higher elevations near glacial sources, are less affected by other factors. In contrast, downstream regions experience slower surface discharge, flow dispersion, and significant water extraction for irrigation, industrial, and domestic uses, reducing water volume.

These details are now elaborated in Section 3.1.3.

**The supplementary content is as follows:**

Line 314-356: As shown in the figure, between 2000 and 2016, the average annual growth rates of discharge at the K1, K2, and K4 cross-sections were 0.36%, 1.2%, and 0.77%, respectively. This upward trend can be primarily attributed to the accelerated melting of glaciers driven by global warming ^[24]^. In particular, the increased contribution of glacial and snowmelt water from the Tianshan Bogda Mountain area—the primary water source of the Ertanggou Basin—has led to a rise in water input into the basin. This phenomenon has also caused seasonal variations in discharge, with the highest rates occurring during the summer when elevated temperatures accelerate glacier melt, resulting in peak discharge. From 2016 to 2020, the annual discharge at the four cross-sections exhibited a marked overall increase, with average annual growth rates of 1.34%, 3.14%, 3.13%, and 7.71%, respectively. A comparative analysis of the two periods (2000–2016 and 2016–2020) reveals that the average annual growth rates of discharge at the K1, K2, and K4 cross-sections significantly increased after 2016. This trend underscores the substantial role of reservoir operations post-2016 in enhancing cross-sectional discharge. In July 2018, cross-sectional discharge peaked, primarily due to the impact of climate change. During this period, temperatures in Xinjiang were higher than in previous years, leading to accelerated glacier melt and a considerable increase in flow. However, climate change remains subject to seasonal effects, causing a pronounced peak during summer followed by a gradual decline ^[25]^. Between 2016 and 2020, the monthly discharge at the K1 cross-section was significantly higher than at the other cross-sections, nearly three times greater. This can be attributed to the construction and operation of the Ertanggou Reservoir, which achieved the goal of river interception, effectively regulated downstream flow, ensured sufficient water availability for downstream ecological needs, and maintained the stability of the river ecosystem. Over the past 20 years, the annual discharge at the K1 cross-section has consistently exceeded that of the downstream cross-sections. This disparity is largely due to geographic factors, as the K1 cross-section is situated at the mountain outlet upstream of the reservoir at a higher elevation, where glacial meltwater directly contributes to river flow. In contrast, as elevation decreases downstream, surface discharge slows, and water flow becomes more dispersed. Additionally, the downstream region, characterized by dense populations and intensive agricultural activity, diverts substantial water resources for irrigation, industrial, and domestic use, further reducing water reserves. Since 2016, following the completion of river channel regularization projects, the monthly discharge at the downstream K2, K3, and K4 cross-sections has shown a slight declining trend. However, the fluctuations have been minimal, with discharge levels remaining relatively stable. This demonstrates that the implementation and optimization of water conservancy projects can significantly reduce water loss during conveyance and increase the volume of water available for downstream use.

24.Wang Puyu, Li Zhongqin, Zhou Ping, Jin Shuang, & Chen Hui. (2014). Recent changes in representative glaciers in Hami, Xinjiang, and their impact on water resources. Advances in Water Science (04), 518-525. doi: 10.14042/j.cnki.32.1309.2014.04.009.

25.Xinjiang Meteorological Bureau. (2019). 2018 Xinjiang Climate Bulletin and Impact Assessment. Urumqi Evening News. Available at: <https://www.btzx.com.cn/web/CHAL1492477520535104/2019/1/22/ARTI1548146889983739.html>

**Comment3:** Similarly, the average annual ecological water conveyance was 0.61×10^8^m^3^, with an efficiency of 10%, 2% higher than the previous 15 years. What is the justification/reason for this change; Since no drastic discharge changes has-been observed after 2010 before 2010 that has occurred, but it is not correlating with discharge changes in subsequent -the linkage between each parameter and their statistical significance has not been discussed clearly and it needs further discussion and revision.

**Answer:** Thank you for your query regarding the increase in ecological water conveyance efficiency from 8% to 10% following the completion of the Ertanggou Reservoir and artificial channel construction in 2014. This improvement can be attributed to several key factors:

**1.Reservoir Development:** The establishment of the Ertanggou Reservoir in 2014 and the subsequent restoration of downstream river channels significantly enhanced water management capabilities within the basin. This allowed for more effective regulation of water flow, reducing losses from uncontrolled leakage and evaporation.

**2.Optimized Water Resource Allocation:** Improved infrastructure facilitated efficient allocation and utilization of water resources, ensuring a greater volume of water reached downstream residential and ecological areas. Reduced biological water loss increased the water available for ecological purposes, boosting conveyance efficiency.

**3.Climate Considerations:** Despite a decrease in precipitation and water reserves in 2018 due to Xinjiang’s arid climate, the enhanced management system maintained a relatively stable efficiency of approximately 10%, representing a clear improvement over prior years ^[26]^.

These factors are discussed in detail in Section 3.2.1 of the manuscript. We hope this explanation sufficiently justifies the observed improvement in conveyance efficiency. Simultaneously, under We acknowledge this important point. The stable discharge trends after 2010 reflect the natural variability of glacial meltwater and the influence of reservoir operations. The significant changes observed after 2016 are closely linked to the completion of the Ertanggou Reservoir.

While this study primarily focuses on the patterns of discharge changes and ecological water conveyance benefits, we recognize the need for a more detailed analysis of correlations between parameters. We aim to address this in future research by integrating statistical analyses with hydrological modeling to clarify these relationships comprehensively.

**The supplementary content is as follows:**

Line 367-379: The construction of the reservoir has been the primary factor influencing this trend. In 2014, during the construction of the Ertanggou Reservoir and the downstream river channel regularization and rectification, a significant amount of ecological water was lost, leading to a substantial reduction in the water volume reaching the K4 section. However, between 2015 and 2020, following the completion of the reservoir and artificial channel construction in November 2014 and the gradual improvement of river channel regularization efforts in 2015, water resources were allocated and utilized more effectively. During this period, the discharge at the two sections exhibited a statistically significant increasing trend (P < 0.05). Furthermore, the average annual ecological water conveyance volume rose to 0.61 × 10⁸ m³, while the water conveyance efficiency exceeded 10%.

26. Zhang Huiqin, Du Dongsheng, Fan Ze, & Ma Hong. (2024). Study on Vegetation Change in Turpan City and Its Response to Climate Change. Inner Mongolia Meteorology (02), 39-43. doi: 10.14174/j.cnki.nmqx.2024.02.007.

**Comment4:** The annual or seasonal discharge rates are also to be discussed here that are influenced by precipitation, snowmelt, groundwater contributions, human activity, and of course climate change. Details of intervention affecting the discharge like hydropower plants, reservoir and dam etc., are to be linked in the discussion. The Climate Change influenced by environmental aspects are also to be analyzed and linked using hydrological modeling, ecosystem changes, change in sediment transport and river morphology, impact of agriculture actives, etc. **Answer:** Thank you for highlighting the importance of discussing annual and seasonal discharge rates. We have expanded the discussion section to include a more comprehensive analysis of factors affecting discharge, such as temperature, elevation, human activity, and interventions like reservoirs and wetland preservation.

In the upstream region, annual discharge is primarily influenced by temperature-driven glacial meltwater, with minimal loss due to high elevation and fewer external impacts. However, as elevation decreases, surface discharge slows, flow disperses, and groundwater infiltration occurs, reducing water volume downstream. Downstream areas, being densely populated and agriculturally active, see significant water extraction for irrigation, industrial, and domestic uses, further depleting water reserves.

While this study does not focus on precipitation effects—given that the region's agriculture relies on artificial irrigation—we recognize its relevance and have noted its potential influence. Additionally, we have detailed the role of interventions like fish ladders and wetland preservation in conserving water and supporting long-term environmental health. These measures offer valuable insights for similar projects in other regions.

Finally, we acknowledge the need for a detailed analysis of the factors contributing to discharge changes. As you suggested, future research will employ hydrological models, adjust temporal and spatial scales, and examine changes in river morphology to address these limitations.

**The supplementary content is as follows:**

Line 458-481: This study focuses on the Ertanggou Basin, an arid region in Xinjiang, analyzing discharge at four cross-sections within the basin. Results reveal that areas reliant on glacial meltwater as their primary source of water exhibit annual discharge directly influenced by temperature. High-altitude regions experience minimal water loss, resulting in substantial annual discharge. However, as elevation decreases, surface discharge velocity slows, water flow diverges, and a portion infiltrates into the ground, leading to reduced water volume downstream. In the downstream oasis areas, characterized by dense populations and frequent agricultural activities, significant amounts of water are diverted for irrigation, industrial, and domestic use, further depleting reserves. This study does not account for interannual variations in precipitation or its impact on assessing river discharge and ecological water conveyance benefits. This omission is justified, as increased precipitation, while increasing available water in the basin, is not a critical factor for the arid Ertanggou Basin. Instead, the quantity of ecological water conveyance plays a decisive role in influencing downstream discharge. Agricultural development in the irrigation areas remains reliant on artificial water diversion, while oases are primarily distributed around rivers and low-lying regions where groundwater naturally seeps out ^[27-28]^. Additionally, this study emphasizes discharge change patterns and the impacts of ecological water conveyance on land-use areas downstream but lacks a detailed analysis of the various factors contributing to these impacts. Consequently, the research does not sufficiently quantify the contribution rates of individual factors to discharge variations. Future research will address this gap by employing hydrological models ^[29]^, altering temporal and spatial scales ^[30-31]^, and analyzing changes in river morphology.

27.Qiao Peng & Wang Lan. (2020). Estimation of Water Resource Availability and Analysis of Water Consumption Balance in the Ertanggou Small Watershed Based on Evapotranspiration. Shaanxi Water Resources (11), 26-28+32. doi: 10.16747/j.cnki.cn61-1109/tv.2020.11.008.

28.Xin Zhang. Characteristics and Influencing Factors of River discharge Changes in the Hami Region of Xinjiang (Doctoral dissertation, Northwest Normal University).

29.Li Huazhen, Zhang Qiang, Gu Xihui & Shi Peijun. (2018). The Impact of Climate and Agricultural Planting Area Changes on discharge in the Yellow River Basin. Journal of Lake Sciences (04), 1138-1151.

30.Fu BJ, Xu YD, Lv YH. Scale characteristics and coupled research of landscape pattern and soil and water loss. Advances in Earth Science, 2010, 25 (7): 673-681.

31.Savary S, Rousseau AN, Quilbe R. Assessing the effects of historical land cover changes on discharge and low flows using re mote sensing and hydrological modeling. Journal of Hydrologic Engineering, 2009, 14 (6): 575-587.

**Comment5:** Since they are taking about the environmental flow impacts on nutrient loading for nutrient regulations is very important that has also to be brought out here as well. The benefits and drawbacks of this ecological water conveyance are critical and are not addressed which will affect the soil fertility, biodiversity preservation, habitat maintenance, natural filtration of pollutants and nutrients and adaptation to climate change. They should bring out how this will help to mitigate these changes that are happening that will affect the ecosystems resilience that are altered to changes in flow conditions. This will reduce/enhance the vulnerability to climate impacts by balancing human and ecological needs of river water. Next from their observation can they bring out how this will help to make decisions on how much water can be safely extracted for human use without damaging ecosystems and for sharing by multiple users and enhance input for river and wetland restoration and other projects.

**Answer:** Thank you for emphasizing the importance of nutrient loading and its regulation in the context of environmental flow impacts. We have added a new section to the manuscript discussing the ecological significance and challenges of water conveyance in arid and semi-arid regions.

Ecological water conveyance plays a crucial role in restoring wetland and river ecosystems, improving soil fertility, preserving biodiversity, and facilitating natural pollutant filtration. However, it also poses challenges, including water quality issues, soil salinization, ecological disturbances, and resource competition. To address these, we propose strengthening water quality monitoring and treatment to ensure conveyed water meets environmental standards and reduces ecosystem pollution. Additionally, optimizing water conveyance design to emulate natural systems can help maintain ecological balance.

We also discuss measures to manage soil fertility and salinization, such as rotational and intermittent irrigation, which prevent soil degradation and enhance resilience. Using natural filtration systems like wetlands in combination with agricultural management can effectively reduce pollution sources and nutrient discharge.

These revisions provide a balanced view of the benefits and drawbacks of ecological water conveyance, offering actionable strategies for mitigating risks while supporting ecosystem resilience and sustainable development.

We believe that these additions address your concerns and provide a more comprehensive understanding of the ecological implications of water conveyance.

**The supplementary content is as follows:**

Line 566-584: Ecological water conveyance plays a crucial role in improving the ecological environment of arid and semi-arid regions. It helps restore wetland and river ecosystems, enhances soil fertility, and promotes biodiversity conservation while supporting the natural filtration of pollutants. However, ecological water conveyance also presents challenges, such as potential water quality issues, ecological disturbances, soil salinization, and competition for limited water resources. To maximize the benefits of ecological water conveyance while mitigating its negative impacts, several solutions must be implemented. First, strengthening water quality monitoring and treatment is essential to ensure that water quality meets environmental standards and reduces ecological pollution. Second, the design of water conveyance systems should be optimized to mimic natural water systems, minimizing disruption to the existing ecological balance. Additionally, soil fertility and salinization management should be prioritized, with measures such as rotational irrigation and intermittent irrigation to prevent soil salinization and enhance soil resilience. Natural filtration systems, such as wetlands, can be used in combination with agricultural management practices to manage pollutants and nutrients, thus reducing pollution sources. Finally, in response to climate change, flexible water conveyance plans should be developed to enhance the resilience and recovery capacity of ecosystems.

**Comment6：**They have not brought out the linkage between the long-term changes impact of glacier mass and melt rate, shift in peak discharge periods and timing and resulted long-term decline in glacier mass and water availability in future.

**Answer:** Thank you for emphasizing the importance of long-term strategies to enhance water conveyance efficiency and mitigate ecological risks.

In the revised manuscript, we address the balance between the benefits and challenges of ecological water conveyance, including its impact on water quality, soil fertility, and biodiversity. To mitigate these challenges, we propose several strategies. For instance, adaptive water management practices, such as rotational and intermittent irrigation, can reduce soil salinization and improve resilience. We also highlight the role of natural filtration systems, such as wetlands, in regulating nutrients and improving water quality, which are essential for ecological stability.

Additionally, we discuss optimizing the spatial and seasonal distribution of water resources to balance human and ecological needs, reducing ecosystems’ vulnerability to climate change while supporting the resilience of river and wetland environments.

Drawing on our observations, we propose establishing safe water extraction thresholds to ensure sustainable human use while maintaining ecological functions. These findings can inform the development of river and wetland restoration projects and serve as a valuable reference for similar arid regions worldwide.

**The supplementary content is as follows:**

Line 522-555: Analyzing the spatiotemporal dynamics of ecological water diversion in the Ertanggou Watershed reveals an overall increasing trend in ecological water diversion from 2016 to 2020. However, pronounced imbalances in seasonal and spatial distribution persist. These dynamics are influenced by climate change, ecological water demand patterns, and long-term changes in glacier mass and meltwater rates. This is consistent with the research conclusions of other scholars at present. Among them, Zhang Hui et al. ^[41]^ analyzed long-term observational data from the Kuitun River Basin in the Tianshan Mountains of Xinjiang and found that over the past 20 years, glacier mass in the basin has steadily decreased, and the melt rate has accelerated, resulting in increased summer discharge. In contrast, winter discharge has decreased due to a reduction in glacial meltwater. This seasonal variation in glacier melt directly influences the spatiotemporal dynamics of ecological water conveyance. Similarly, Li ZH et al. ^[42]^ examined the discharge response of the Loess Plateau in China to environmental changes and found that, due to the long-term decline in glacier mass, the timing of peak flow has shifted. The traditional summer peak has occurred earlier, while winter flows have decreased, further exacerbating the imbalance in water distribution between upstream and downstream areas. The spatial variability in ecological water diversion underscores the complexity and regional differences inherent in the process. This suggests the necessity of optimizing water diversion strategies based on the ecological needs of different seasons and regions to ensure ecosystem stability and sustainable development ^[43]^. Maintaining a stable forest and grassland landscape while enhancing the watershed’s water conservation capacity is also critical to sustaining the ecological water sources of the basin ^[44]^. Future research on ecological water diversion strategies should study and predict the long-term impact of diminishing glacier mass and changing meltwater rates to adapt to these evolving challenges ^[45]^. Water diversion strategies must be adjusted to account for shifts in peak flow periods, ensuring that ecological water needs are met throughout different seasons. Strengthening the balance of water resource distribution between upstream and downstream areas through storage and regulation facilities, coupled with advanced technologies to optimize spatial water distribution, will also be essential. Furthermore, improving the watershed’s water conservation capacity by protecting and restoring forest and grass vegetation can enhance the basin’s water resource regulation functions, providing a scientific basis for sustainable water resource management ^[46]^.

41.Zhang Hui, Li Zhongqin, Mou Jianxin, & He Haidi. (2017). Glacier changes in the Kuitun River Basin of the Tianshan Mountains in Xinjiang over the past 50 years and their impact on water resources. Geographical Science (11), 1771-1777. doi: 10.13249/j.cnki.sgs.2017.11.020.

42.LiZ H, Wang Y M, Zhang H B, Chang X, Yu Y H. discharge response to changing environment in Loess Plateau, China: implications of the influence of climate, land use/land cover, and water withdrawal changes. Journal of Hydrology, 2022, 613: 128458

43.Wang Yongpeng. (2022). Study on the Ecological Water Diversion Effect and Water Allocation Strategy in the Lower Reaches of the Tarim River (Master's Thesis, Xinjiang Agricultural University). Master's Thesis. Available at: https://link.cnki.net/doi/10.27431/d.cnki.gxnyu.2022.000202 doi: 10.27431/d.cnki.gxnyu.2022.000202.

44.Jin X T, Zhi X T, Jun B L, 0in 2 H. Contribution of changes in vegetation composition and climate variability on streamflow across the global watersheds. Catena,2023, 232:107394.

45.Hayat Huma, Tahir Adnan Ahmad, Wajid Sara, Abbassi Arshad Mehmood, Zubair Fatima, Hashmi Zia ur Rehman... & Irshad Muhammad. (2019). Simulation of the meltwater under different climate change scenarios in a poorly gauged snow and glacier-fed Chitral River catchment (Hindukush region).Geocarto International(1),1-17.

46.Zhi Liang, Ruochen Sun & Qingyun Duan. (2024). Attribution of Vegetation Dynamics in the Yellow River Water Conservation Area Based on the Deep Conv LSTM Model. Remote Sensing (20),3875-3875.

**Response letter to Reviewer#2**

**(our responses are in blue color and the actions in reviewed manuscript are in red color)**

Thank you for your thoughtful and constructive feedback on our manuscript. Your comments have been invaluable in helping us improve the quality of our paper. Below, we provide detailed responses to each comment and highlight the corresponding revisions made in the manuscript.

**Comment1**: Add the background of the study in the abstract.

**Answer:** We agree with your suggestion and have reorganized the abstract to include the background of the study. This addition provides readers with a clearer understanding of the study's context. The revised abstract now contains the study background on lines 9–12 of the manuscript.

**The supplementary content is as follows:**

Line 9-12: Given the current ecological degradation in the inland arid river basins of our country, ecological water conveyance has increasingly become a critical measure to alleviate the pressures of water scarcity on ecosystems and to promote regional sustainable development.

**Comment2**: It is necessary to specify what the project is for ecological water conveyance in the study area, as many international readers are not aware of the context of the study, consider placing it in the introduction or in the overview of the study map.

**Answer:** Thank you for pointing this out. To provide international readers with a better understanding of the ecological water conveyance project, we have included an introduction to the project in the "Overview of the Study Area" section. The additional information is now presented on lines 118–136 of the manuscript.

**The supplementary content is as follows:**

Line 120-138: In recent years, water resource shortages in the Shanshan County basin have led to significant challenges, including river interruptions, vegetation degradation, and difficulties in securing adequate irrigation water for farmlands in downstream river channels and residential areas ^[17]^. To address the pressing demands of economic and social development and to optimize the allocation of water resources within the basin, the government completed the “Feasibility Study Report on Key Water Resource Allocation Projects in Shanshan County” in 2015. Following this, a series of water-saving transformation measures were implemented. Among these efforts, the construction of the Ertanggou Reservoir began in September 2012. By November 2014, the reservoir was operational, with a total storage capacity of 2.36×108 m^3^ and an irrigation control area extending to 202,600 acres. This reservoir has played a crucial role in alleviating severe water shortages in downstream oasis communities, increasing irrigation water for agricultural fields, and providing a reliable water resource supply for the daily needs of downstream urban residents ^[18]^. By August 2016, the Ertanggou Reservoir had reached its normal storage level. It is located at the third cross-section, which was formed following the completion of river channel regulation and alignment works. However, the absence of suitable hydrological stations makes it difficult to monitor changes in river flow prior to the implementation of ecological water conveyance.

17.Li Yupeng, Chen Yaning, Ye Zhaoxia, Wang Fei, Sun Fan, Qin Jingxiu. Ecological responses to 20 years of water conveyance in the lower reaches of the Tarim River [J]. Arid Zone Geography, 2021, 44(3): 700-707.

18.Zhang Xun. (2018). A Preliminary Study on Water Resource Allocation in Shanshan County. Jilin Water Resources (09), 47-50. doi: 10.15920/j.cnki.22-1179/tv.2018.09.015.

**Comment3**: Explanations of formulas should be topspaced and not indented. Revise line 149 and its counterpart in the manuscript.

**Answer:** Following your suggestion, we have adjusted the formatting of the explanations of formulas to ensure top spacing and removed the indentation. These changes have been applied to line 149 and similar instances in the manuscript.

**Comment4**: The results in Figure 3 are interesting and should be discussed in more detail.

**Answer:** We appreciate your interest in the results presented in Figure 3. Based on your suggestion, we have expanded the discussion of Figure 3. Specifically, we have provided a detailed explanation of the RSHS estimation model and the process used to generate the cross-section shape maps and digital river model maps (lines 275–284). Additionally, we have conducted a more thorough analysis of the results shown in Figure 3 (lines 286–292).

**The supplementary content is as follows:**

Line 275-284: The middle section of the river, as captured by the uncrewed aerial vehicle (UAV), is selected as the cross-section for flow estimation, ensuring that the upstream and downstream distances are approximately equal. For the submerged portions of the riverbed, underwater topography is acquired through direct cross-sectional measurements. Meanwhile, the topography above the water surface is derived from DSM data obtained via UAV surveys. By integrating the UAV-captured terrain data for the above-water portion with the measured underwater data, the complete cross-sectional profile of the river channel is generated through seamless splicing of the upper and lower terrains.

Line 286-292: The digital river channel model on the left effectively represents the three-dimensional structure of the river channel, providing a clear depiction of its shape. In comparison, the cross-sectional profile graph on the right illustrates the water depth and surface width at the selected cross-section. The water level variations simulated by the model align closely with trends observed during field surveys, demonstrating the model’s capability to accurately capture the hydrological processes of the studied river section.

**Comment5**: The font in Figure 7 is too small and somewhat blurred.

**Answer:** Thank you for bringing this to our attention. We have increased the font size in Figure 7 to improve readability and have also enhanced the overall image quality to eliminate any blurring issues. These adjustments ensure the figure is clearer and more visually accessible to readers. As shown in the figure below:

**Figure 7. Correlation analysis of each factor**

**Comment6**: The discussion is too brief and needs to be added. There should be a discussion of the research methodology, the similarities and differences between the results and others. For example, the prospect of the application of this technical method in the arid zone in comparison with other studies, whether there are ecological water transfer projects in other regions and their comparison, and other aspects.

**Answer:** Thank you for your insightful suggestion to expand the discussion section. In response, we have significantly enhanced Section (2) of the discussion by including the following points:

**Comparison of Results:** We have added a subsection comparing our findings with those of other studies. This comparison highlights both consistencies and variations, offering a broader context for understanding our results.

**Application in Arid Zones:** We have discussed the potential application of our technical method in arid zones and compared it with similar studies to illustrate its unique contributions and limitations in such environments.

These additions aim to provide readers with a deeper analysis of our research methodology and findings in comparison to other ecological water transfer projects.

**The supplementary content is as follows:**

Line 483-508: Numerous domestic and international scholars have employed diverse methods to investigate discharge changes across various river basins, yielding significant findings. Many studies have demonstrated that ecological water conveyance can provide additional water sources for rivers, enhancing the economic benefits of downstream agriculture. For example, Ailike Si Laiyin et al. ^[32]^ calculated environmental quality indices to comprehensively evaluate the natural environmental quality of the lower Tarim River. Their findings show that from 2000 to 2020, ecological water conveyance significantly improved the natural environmental quality of the lower Tarim River. Similarly, Wang Chuan et al. ^[33]^ used long-term monitoring and remote sensing data in the Ejina Oasis to analyze hydro-ecological indicators' responses to ecological water conveyance volumes, clarifying the driving factors and intensities influencing vegetation restoration in the oasis. Unlike these previous studies, particularly when compared to large-scale water conveyance projects in China designed to alleviate water scarcity in northern regions ^[34-35]^, the Ertanggou Watershed project is specifically tailored to the ecological requirements of arid regions. This project emphasizes not only water resource storage but also integrates water resource protection and rational allocation to maximize ecological benefits. It underscores the importance of localized solutions when addressing ecological water challenges. During the implementation of the Ertanggou project, the environmental impacts of reservoir construction and the long-term sustainability of the ecological water conveyance efforts were carefully considered ^[36-37]^. Measures such as the construction of fish ladders and the preservation of wetland areas were adopted to minimize environmental disruption ^[38]^. As more regions worldwide face water scarcity, the experience gained from the Ertanggou Watershed project offers valuable insights for developing ecological water conveyance strategies in other arid regions.

32.Ailikemu Silihan, Anwar Aiziz, Tajiguli Kasim, Shi Zhiwen, Xu Junyu, Zewariguli Kebier... & Alisier Kurban. Assessment of the Ecological Water Diversion Benefits in the Lower Reaches of the Tarim River Based on the Environmental Quality Index. Arid Zone Geography, 1-15.

33.Wang Chuan, Zhang Yongyong & Zhao Wenzhi. (2022). The Eco-hydrological Restoration Effects of Ecological Water Diversion in the Ejina Oasis, Lower Reaches of the Heihe River, in the Last Two Decades. Journal of Ecology (11), 2180-2187. doi:10.13292/j.1000-4890.202210.017.

34.Yan, H., Lin, Y., Chen, Q., Zhang, J., He, S., Feng, T., Wang, Z., & Chen, C. (2023). A Review of the Eco-Environmental Impacts of the South-to-North Water Diversion: Implications for Interbasin Water Transfers. Engineering, 30, 161-169. https://doi.org/10.1016/j.eng.2023.05.012

35.Xu Zhangxing, Liu Siyuan, Li Yiwen, Jiang Zheng & Jin Yu. The Impact of the South-to-North Water Diversion Project on the Non-agriculturalization of Water Resources in the Receiving Area. South-to-North Water Diversion and Water Science and Technology (in Chinese and English) 1-16.

36.Wang Sifan, Xu Hui, Ren Yufeng, Ji Daobin, Zhou Huaming & Long Lianghong. Research Progress on the Impact of Reservoir Construction on River Water Temperature and Ecological Environment. Three Gorges Ecological Environment Monitoring 1-29.

37.Chen Nuo, Bao Dan & Zhu Zhilin. (2024). Discussion on the Ecological Environment Construction of Wangjiachong Reservoir Area in the Water Resources Allocation Project of Northern Hubei. Theoretical Research in Urban Construction (Electronic Edition) (13), 202-204. doi: 10.19569/j.cnki.cn119313/tu.202413067.

38.Li Jiang, Liu Ying, Wu Tao & Peng Zhaoxuan. (2020). Seventy Years of Reservoir Dam Construction Achievements in Xinjiang. Journal of the China Institute of Water Resources and Hydropower Research (05), 322-330. doi: 10.13244/j.cnki.jiwhr.20200501.

**Comment7**: Overall, the research is solid, but the writing of the manuscript should continue to improve. In particular, the title of the manuscript is based on the study of ecological water transfer, for readers in other regions do not understand what period of time the project began to realize its benefits, and then there is some lack of understanding of the assessment of its benefits.

**Answer:** Thank you for your positive feedback and constructive suggestions. We are pleased that you found the research solid and have taken steps to further improve the manuscript’s clarity and accessibility.

**Title Revision:** To provide readers with a clearer understanding of the study’s timeline, we have revised the title to: "Estimation of Long-Term River Discharge and Benefits Evaluation of Ecological Water Conveyance in Ertanggou Basin from 2000 to 2020."

**Expanded Content:** We have added details in the "Study Area" and "Results" sections to outline the timeline of the project’s implementation and the stages at which its benefits became evident.

**Benefit Assessment:** A new subsection has been included in the discussion to specifically evaluate the project’s benefits. This subsection explains the criteria used for assessment and aligns these benefits with the project’s objectives.

These revisions ensure that the manuscript is more informative and accessible to a global audience, particularly readers unfamiliar with the context of ecological water transfer projects.

We appreciate your valuable feedback and believe that these revisions have significantly enhanced the quality and comprehensibility of our manuscript. Thank you for your time and effort in reviewing our work.
