# Supplementary material for: Estimation of long-term river discharge and benefits evaluation of ecological water conveyance in Ertanggou basin from 2000 to 2020
Source: PLoS One. 2025 May 9;20(5):e0321990. doi: 10.1371/journal.pone.0321990 (PMC12063809; doi:10.1371/journal.pone.0321990)
Supplement: Attachment — Submitted filename: Response_to_Reviewers_auresp_2.docx [file pone.0321990.s003.docx]

To Editor:

Thank you for your letter and the constructive comments on this article in your busy schedule. All of us authors have carefully read the comments that you have given us, and have discussed and revised each of these issues. The following is my list of revisions. In addition, we have resubmitted a new manuscript in the revised state, with the revisions highlighted in red. If there are any incorrect answers or questions in the manuscript, please do not hesitate to let us know.

Kind regards,

Adilai Wufu

**Response letter to Reviewer 1**

**(Our responses are in blue color and the actions in reviewed manuscript are in red color)**

Thanks a lot for your time and effort to improve our manuscript. The authors really appreciate your thoughtful and favorable opinions on our manuscript. We have considered carefully about your detailed comments, questions and opinions, and the manuscript was revised accordingly.

**Comment:** They have addressed most of all the queries raised by the reviewers by some are not answered clearly like what period of time the project began to realize its benefits, and then there is some lack of understanding of the assessment of its benefits. it may be ignored.

**Answer:** We appreciate your thorough review of our manuscript and the meticulous attention to detail in your comments. In response to your queries, we would like to provide the following clarifications:

Regarding the timeline of project benefits, this information is included in the study area overview. The project milestones are as follows: reservoir construction began in 2012, operational storage commenced in 2014, a regional water resources allocation feasibility meeting was held in 2015, and river regulation works were completed in 2016. These milestones outline the timeframe within which the project began yielding benefits.

**The related content is as follows:**

**Line 123-134:** To address the pressing demands of economic and social development and to optimize the allocation of water resources within the basin, the government completed the “Feasibility Study Report on Key Water Resource Allocation Projects in Shanshan County” in 2015. Following this, a series of water-saving transformation measures were implemented. Among these efforts, the construction of the Ertanggou Reservoir began in September 2012. By November 2014, the reservoir was operational, with a total storage capacity of 2.36×108 m^3^ and an irrigation control area extending to 202,600 acres. This reservoir has played a crucial role in alleviating severe water shortages in downstream oasis communities, increasing irrigation water for agricultural fields, and providing a reliable water resource supply for the daily needs of downstream urban residents ^[18]^. By August 2016, the Ertanggou Reservoir had reached its normal storage level.

18. Zhao Fuyong. Hydrological calculation and analysis of the Ertanggou River in Xinjiang under engineering regulation [J]. Water Conservancy Science and Cold Region Engineering, 2021, 4(04): 139-142.

Regarding the assessment of the project's benefits, we have analyzed the achievements of the Ertanggou project in the comparative analysis section, emphasizing its role in water resource allocation and protection. Additionally, we have incorporated a summary in the conclusion, as per your suggestion, quantifying the benefits of ecological water conveyance. Our findings indicate that the project has enhanced both the volume and efficiency of ecological water conveyance in the Ertanggou River Basin, leading to an increase in water surface area and agricultural land, thereby demonstrating its positive outcomes.

We have carefully considered your feedback and made the necessary revisions to improve the clarity and robustness of our manuscript.

**The related content is as follows:**

**Line 499-513:** Unlike these previous studies, particularly when compared to large-scale water conveyance projects in China designed to alleviate water scarcity in northern regions ^[35-36]^, the Ertanggou Watershed project is specifically tailored to the ecological requirements of arid regions. This project emphasizes not only water resource storage but also integrates water resource protection and rational allocation to maximize ecological benefits. It underscores the importance of localized solutions when addressing ecological water challenges. During the implementation of the Ertanggou project, the environmental impacts of reservoir construction and the long-term sustainability of the ecological water conveyance efforts were carefully considered ^[37-38]^. Measures such as the construction of fish ladders and the preservation of wetland areas were adopted to minimize environmental disruption ^[39]^. As more regions worldwide face water scarcity, the experience gained from the Ertanggou Watershed project offers valuable insights for developing ecological water conveyance strategies in other arid regions.

35.Yan, H., Lin, Y., Chen, Q., et al. (2023). A Review of the Eco-Environmental Impacts of the South-to-North Water Diversion: Implications for Interbasin Water Transfers. Engineering, 30, 161-169. https://doi.org/10.1016/j.eng.2023.05.012

36.Xu Zhangxing, Liu Siyuan, Li Yiwen, Jiang Zheng & Jin Yu. The Impact of the South-to-North Water Diversion Project on the Non-agriculturalization of Water Resources in the Receiving Area. South-to-North Water Diversion and Water Science and Technology (in Chinese and English) 1-16.

37.Wang Sifan, Xu Hui, Ren Yufeng, et al. Research Progress on the Impact of Reservoir Construction on River Water Temperature and Ecological Environment. Three Gorges Ecological Environment Monitoring 1-29.

38.Chen Nuo, Bao Dan & Zhu Zhilin. (2024). Discussion on the Ecological Environment Construction of Wang jia chong Reservoir Area in the Water Resources Allocation Project of Northern Hubei. Theoretical Research in Urban Construction (Electronic Edition) (13), 202-204. doi: 10.19569/j.cnki.cn119313/tu.202413067.

39.Li Jiang, Liu Ying, Wu Tao & Peng Zhaoxuan. (2020). Seventy Years of Reservoir Dam Construction Achievements in Xinjiang. Journal of the China Institute of Water Resources and Hydropower Research (05), 322-330. doi: 10.13244/j.cnki.jiwhr.20200501.

**The supplementary content is as follows:**

**Line 617-620:** On the premise that river training and confinement projects significantly reduce losses along the ecological water conveyance, the ecological water conveyance can provide sufficient water resources for the downstream wetland as well as the downstream river perimeter.

**Line 625-628:** The implementation of ecological water diversion has not only improved the ecological environment of the river basin but also promoted agricultural development by enhancing the efficiency of water resource utilization.

**Response letter to Reviewer 2**

**(our responses are in blue color and the actions in reviewed manuscript are in red color)**

Thanks a lot for your time and effort to improve our manuscript. The authors really appreciate your thoughtful and favorable opinions on our manuscript. We have considered carefully about your detailed comments, questions and opinions, and the manuscript was revised accordingly.

Please see our point-to-point responses below.

**Comment1:** Table 1, GEE is generally capitalized.

**Answer1:** The "Gee" in Table 1 has been changed to "GEE".

**Comment2:** Line 169, where initials need to be lowercase here.

**Answer2:** The first word in line 169, "Where" has been changed to "where".

**Comment3:** The formatting of references needs to be improved.

**Answer3:** Thank you for your observation regarding the formatting of the references. I have carefully reviewed and made the necessary adjustments to ensure that the references now adhere to the journal's formatting requirements.

**Comment4:** The methods section could consider adding citations. (https://doi.org/10.1007/s13201-023-02099-7)

**Answer4:** Regarding your suggestion to strengthen the methods section with additional citations, we have reviewed the reference you provided (<https://doi.org/10.1007/s13201-023-02099-7>) and recognize its value in analyzing runoff changes and predicting water resource trends. Given our paper’s structure, we have incorporated this reference in the discussion section, where we compare our findings with existing studies. We also intend to explore its insights further in future research to optimize our experimental design.

Thank you again for your valuable suggestions and support.

**The supplementary content is as follows:**

**Line 494-499:** Xinchen Gu et al. constructed a SWAT hydrological model for the runoff-producing area of the Manas River Basin, analysed the changes in hydrological elements, and predicted the future trend of water resources based on the CMIP5 model, indicating that in the Northwest Arid Zone, the future fluctuation range of water resources is large, and it is necessary to strengthen the monitoring and management of water resources.

34.Gu, X., Long, A., He, X. et al. Response of runoff to climate change in the Manas River Basin flow-producing area, Northwest China. Appl Water Sci 14, 43 (2024). https://doi.org/10.1007/s13201-023-02099-7
